# CtBP—a targetable dependency for tumor-initiating cell activity and metastasis in pancreatic adenocarcinoma

**DOI:** 10.1038/s41389-019-0163-x

**Published:** 2019-10-04

**Authors:** Ayesha T. Chawla, Kranthi Kumar Chougoni, Prashant J. Joshi, Agnes D. Cororaton, Patrick Memari, John C. Stansfield, Haemin Park, Rashmi Seth, Barbara Szomju, Adam P. Sima, Michael O. Idowu, Keith C. Ellis, Steven R. Grossman

**Affiliations:** 1 C. Kenneth and Diane Wright Center for Clinical and Translational Research, Virginia Commonwealth University, Richmond, VA 23298 USA; 2 Department of Internal Medicine, Virginia Commonwealth University, Richmond, VA 23298 USA; 3 Department of Pathology, Virginia Commonwealth University, Richmond, VA 23298 USA; 4 Department of Biostatistics, Virginia Commonwealth University, Richmond, VA 23298 USA; 5 Department of Surgery, Virginia Commonwealth University, Richmond, VA 23298 USA; 6 VCU Massey Cancer Center, Virginia Commonwealth University, Richmond, VA 23298 USA; 7 Department of Medicinal Chemistry, Virginia Commonwealth University, Richmond, VA 23298 USA

**Keywords:** Pancreatic cancer, Cancer stem cells, Target validation

## Abstract

*Ctbp2* is a uniquely targetable oncogenic transcriptional coregulator, exhibiting overexpression in most common solid tumors, and critical to the tumor-initiating cell (TIC) transcriptional program. In the “CKP” mouse pancreatic ductal adenocarcinoma (PDAC) model driven by mutant *K-Ras*, Ctbp2 haploinsufficiency prolonged survival, abrogated peritoneal metastasis, and caused dramatic downregulation of c-Myc, a known critical dependency for TIC activity and tumor progression in PDAC. A small-molecule inhibitor of CtBP2, 4-chloro-hydroxyimino phenylpyruvate (4-Cl-HIPP) phenocopied *Ctbp2* deletion, decreasing tumor burden similarly to gemcitabine, and the combination of 4-Cl-HIPP and gemcitabine further synergistically suppressed tumor growth. Pharmacodynamic monitoring revealed that the 4-Cl-HIPP/gemcitabine combination induced robust and synergistic tumor apoptosis and marked downregulation of the TIC marker CD133 in CKP PDAC tumors. Collectively, our data demonstrate that targeting CtBP represents a fruitful avenue for development of highly active agents in PDAC that cooperate with standard therapy to limit both primary and metastatic tumor burden.

## Introduction

Human pancreatic ductal adenocarcinoma (PDAC) remains among the most lethal of all human solid tumors, with a 93% case fatality rate^1^. Despite modest improvement in survival outcomes with the advent of adjuvant postoperative chemotherapy, and improved survival in stage IV disease with newer multi-agent chemotherapy regimens, the lack of recurring targetable driver oncogene mutations has led to frustratingly slow progress in advancing PDAC therapy. The most notable recurring genetic alterations in PDAC include near-universal K-RAS mutation, along with the frequent loss or mutation of tumor suppressors p53, SMAD4, and CDKN2A^2^. As none of these alterations have yet yielded a robust therapeutic avenue, we considered whether an emerging oncogene in human solid tumors, the uniquely targetable transcriptional coregulator C-terminal Binding Protein (CtBP)^3^, may contribute to PDAC initiation or progression, and if so, whether targeting CtBP in a PDAC mouse model could yield improved outcomes over standard therapy alone.

The paralogous transcriptional coregulators CtBP1 and 2 play a pivotal role in maintaining cellular homeostasis by participating in normal cell functions such as apoptosis^4^, proliferation^5^, as well as exit from pluripotency^6,7^. As a result, inappropriate CtBP overexpression in an otherwise normal cell is associated with oncogenic transformation^8^. In human cancer, CtBP overexpression is strongly correlated with poor prognosis, especially in breast cancer as well as ovarian cancer^9,10^. Mouse studies suggest that the overexpression of CtBP in human cancer is likely causally linked to poor prognosis, as we have shown that in mice mutated for *Apc* (“*Apc min”*) that otherwise die within a few months from extensive intestinal polyposis, *Ctbp2* haploinsufficiency significantly prolongs survival and reduces polyposis by 90%^8^. In addition, we have also recently reported that the rescue of polyposis by *Ctbp2* haploinsufficiency in *Min* mice is due to decreased abundance of tumor-initiating cells in the intestine^11^. This suggests that Ctbp2 plays a crucial role in the transformation from normal stem cells to the tumor-initiating cell phenotype.

Aside from its oncogenic capabilities, CtBP, uniquely among transcription factors, encodes a targetable dehydrogenase domain required for oligomerization and transcriptional function. We have developed a novel class of anti-CtBP therapeutics based on 2-hydroxy-imino phenylpyruvate (HIPP), a CtBP dehydrogenase substrate competitive inhibitor^12^, and HIPP and higher-potency derivatives (4-Cl-HIPP)^12^, exhibit on-target inhibition of CtBP and phenocopy *Ctbp2* haploinsufficiency in reducing polyposis in the *Min* mouse^8^.

In this report, we studied the expression of CtBP1 and 2 in a series of human PDAC specimens and noted universally high expression of both proteins, as seen in other high-grade malignancies, such as high-grade ovarian cancer^10^. *Ctbp2* haploinsufficiency prolonged overall survival and abrogated peritoneal metastasis in an aggressive mutant *K-Ras*-driven mouse PDAC model (CKP), with concomitant downregulation of the proto-oncogene and TIC marker c-Myc. Moreover, therapeutic targeting of CtBP2 in CKP mice with the small-molecule CtBP inhibitor 4-Cl-HIPP, resulted in reduced tumor burden similar to that induced by standard gemcitabine, with the combination synergistically attenuating tumor growth. Finally, we show that combination therapy strongly induced apoptosis in CKP tumors, and that CD133 cancer stem cell marker expression was ablated in mice receiving gemcitabine/4-Cl-HIPP therapy. Our work therefore supports CtBP as a critical and therapeutically targetable dependency in PDAC.

## Results and discussion

**Fig. 1:**
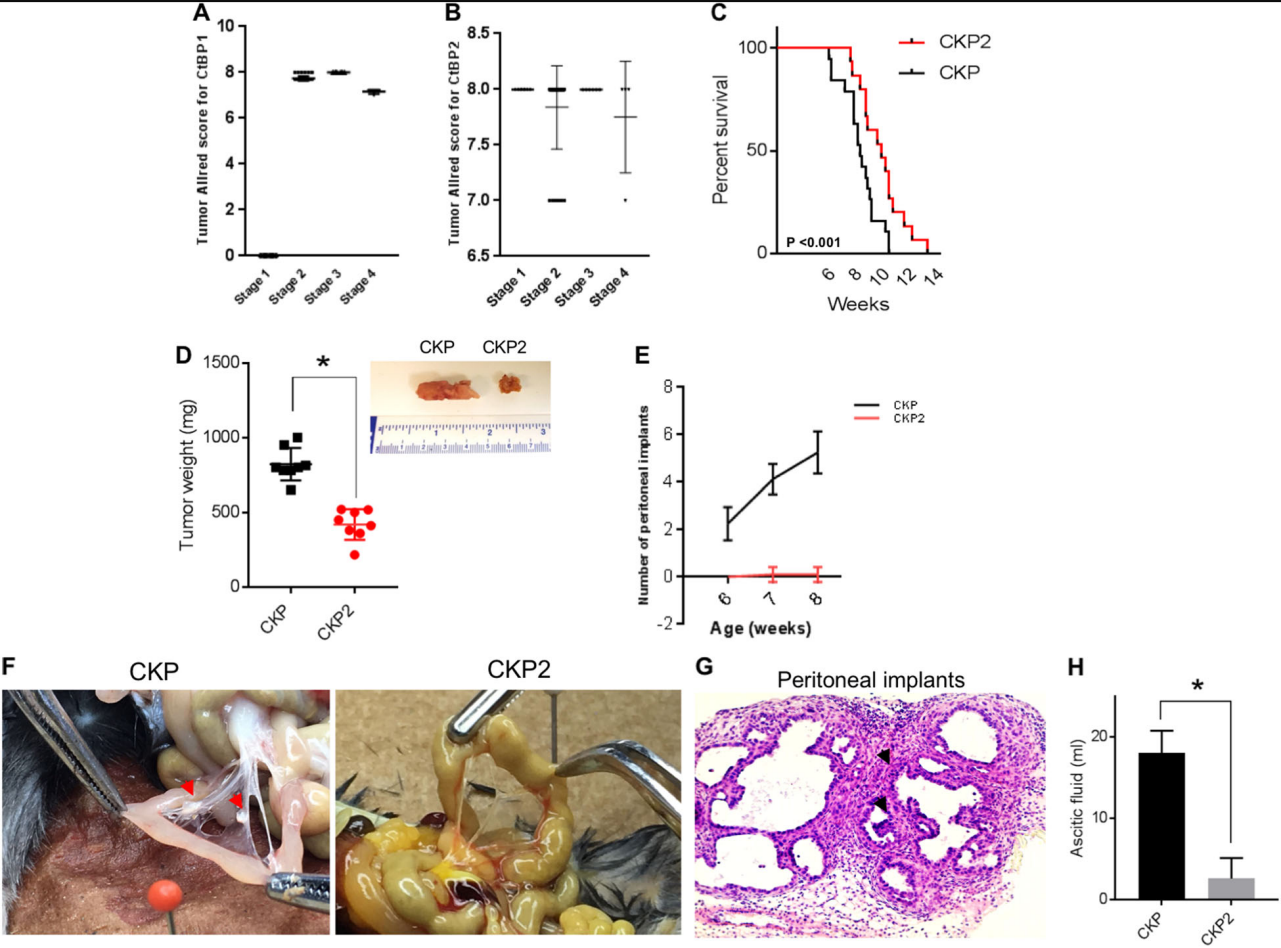
Expression and role of CtBP in human and murine PDAC tumor progression. **a**, **b** Allred score distribution for CtBP1 (**a**) and CtBP2 (**b**) in PDAC tumor specimens obtained from PDAC patients across TNM stages I–IV and stained for CtBP1 and 2 by IHC. **c** Kaplan–Meier survival curves for CKP and CKP2 mice, *n* = 15/group; median survivals were 8.1 and 9.5 weeks, respectively; *p* < 0.001. **d** Box plot of tumor weights obtained from CKP and CKP2 mice, *n* = 10/group; *p < 0.001. (inset) Representative images of CKP vs. CKP2 tumors. **e** Quantitation of the number of malignant peritoneal implants in CKP and CKP2 mice at 6–8 weeks of age. **f** Mesentery of representative CKP and CKP2 mice. Arrows indicate peritoneal implants in CKP mesentery (left panel), while no implants are observed in CKP2 mesentery (right panel). **g** H&E section of metastatic lesion obtained from a CKP mesenteric implant; arrows indicate pleomorphic nuclei (×400). **h** Quantitation of ascitic fluid volumes in each genotype; *p < 0.02. Methods: An ATA27 automated tissue microarrayer was used to construct tissue microarrays (TMAs) that were prepared from tumor-containing portions of pancreatic resection samples of 64 patients identified in the VCU Health Pathology archives with a diagnosis of PDAC, and the TMAs were analyzed for CtBP1 and CtBP2 protein abundance by using IHC. All retrospective human tumor material and clinical information was obtained under a VCU Institutional Review Board approved protocol. For IHC, tumors derived from pancreatic cancer patients were formalin fixed and paraffin embedded on slides, and were subjected to deparaffinization and hydration steps followed by quenching and peroxidase reaction steps^23^. Antigen retrieval was performed at pH 6 by using a pressure cooker, followed by blocking for an hour with 5% goat serum and incubation overnight with primary antibodies at a dilution of 1:100 in blocking buffer. Antibodies—CtBP1 (Cat no. 612042, BD Biosciences); CtBP2 (Cat no. 612044, BD Biosciences, Cambridge, MA). After 3 washes in PBS, secondary antibodies (Dako Envision Systems HRP-conjugated anti-mouse or anti-rabbit IgG, Cat no. K4000, Carpinteria, CA, USA) were incubated for 1 h followed by 3 washes in PBS and slide development by using DAB chromogen substrate from Dako Envision Systems (Cat no. K3467). Nuclear staining was performed by incubation in hematoxylin (Electron Microscopy Sciences, Hatfield, PA, USA) for 2 min and was followed by dehydration with gradient alcohols and coverslipping steps. For the TMA-staining analysis, the Allred scoring system^24^ was used with protein expression rated on a scale from 0 to 8, with 8 being the highest level of expression. Scores for each stain were the average of values obtained from two independent pathologists. All animal studies performed for this paper were approved by VCU Institutional Animal Care and Use Committee. *C57BL/6J* male mice heterozygous for the Ctbp2 allele (*Ctbp2*^*+/*−^) were purchased from Jackson Laboratory (Bar Harbor, ME) and mated with wild-type *C57BL/6J* female mice with further backcross of the mutant genotypes to *C57BL/6J* at least 6 generations before initiating experiments. Allele-specific PCR assays were used to genotype for *Ctbp2* deletion (end-point PCR: *Ctbp2*^*+/−*^ Internal Control, F- 5′-CAAATGTTGCTTGTCTGGTG-3′ and R-5′-GTCAGTCGAGTGCACAGTTT-3′, Mutant F-5′-CCAGTGGGGATCGACGGTATC-3′, and Mutant R-5′-CACTCCAACGCAGCACCATC-3′). For pancreatic tumor mouse models, CKP2 mice were generated by breeding *Ctbp2* ^*+/−*^ mice (as above) with C57BL/6J *Pdx-cre; LSL-K-ras; p53* ^*fl/fl*^ mice (CKP^15^). CKP and CKP2 mice were killed at indicated ages, and tumor weights, peritoneal implants, and ascites were measured. Allele-specific PCR assays were performed for Ctbp2 haploinsufficiency as mentioned above

CtBP’s role in PDAC is relatively unexplored; based on CtBP1/2 expression data from other solid tumors with poor prognosis^13^, we hypothesized that CtBP1/2 expression would be elevated in many, if not all, PDAC specimens. We therefore analyzed CtBP1 and 2 protein expression levels by immunohistochemistry (IHC) in a series of deidentified PDAC surgical resection specimens for which age, sex, histology, and clinical stage were available (Table S1A). In our series, 72% of specimens were from patients with stage 2 pancreatic cancer, and the remainder were obtained from cases split equally across stages 1, 3, and 4. There was an even split between males and females, and the mean age at diagnosis was 62.9, with an overall median survival for the entire cohort of 18 months due to the heavy weighting toward stage 2 cases. As determined by IHC and Allred scoring of intensity and uniformity of expression^14^ (see Materials and Methods; scores 3–8 were considered positively expressing specimens), CtBP1 and CtBP2 were found to be widely and highly expressed in tumor cells across all stages of PDAC (Fig. 1a, b; representative samples shown in Fig. S1A, B), except stage 1 where CtBP2 stained strongly in each case while CtBP1 expression was absent. CtBP1 and CtBP2 expression did not have any significant effect on patient survival (hazard ratio = 1.02 95% CI: (0.68, 1.41), *P* = 0.92 and hazard ratio = 0.71 95% CI: (0.37, 1.37), and *P* = 0.31 for CtBP1 and CtBP2, respectively), due to the uniformly high expression of CtBP1 and 2 in malignant ductal cells, as median Allred scores were 7 or 8 for all stages (with the exception of CtBP1 staining in stage 1 cases) (Fig. 1a, b; Table S2). As the tumor sections used for the CtBP1/2 analysis lacked normal pancreatic ducts to establish baseline physiologic expression of CtBP1/2 in normal pancreatic ductal cells, we performed CtBP1/2 IHC on normal human pancreas sections obtained from 3 resection specimens, and noted significantly lower median Allred scores of 3 and 2 for CtBP1 and 2, respectively, when compared with median Allred scores for each tumor stage (Fig. S1; Table S2; Fig. 1). Thus, CtBP1 and 2 are highly expressed in human PDAC, especially stages 2–4, and expression is significantly higher than baseline physiologic expression in normal pancreatic ductal cells.

To determine whether the uniformly high expression of CtBP2 in human PDAC was linked to an oncogenic role, we studied the impact of *Ctbp2* deletion in the CKP PDAC model^15^. These mice, which die at 9–10 weeks from aggressive and disseminated (to peritoneum) adenocarcinoma, were bred with *Ctbp2*^*+/*−^ mice^16^ to generate CKP mice heterozygous for *Ctbp2* knockout (“CKP2”; ref. ^15^). CKP2 mice were visibly smaller than CKP mice (Fig. S2A), and exhibited prolonged overall survival, with median survival of 9.5 weeks for CKP2 vs. 8.1 weeks for CKP mice, *p* < 0.001 (Fig. 1c). This result recalls the increased overall survival of *Ctbp2*^*+/−*^*Apc*^*min/+*^ as compared with *Apc*^*min/+*^ mice, with the relative prolongation in survival in proportion to the expected native survival of the tumor model^8^. Consistent with prolonged survival, CKP2 tumor weights were ~50% less than those observed in CKP mice (Fig. 1d; Fig. S2B).

Remarkably, metastatic peritoneal implants, prominent in CKP, were nearly absent in CKP2 mice (Fig. 1e, f). Indeed, the number of peritoneal implants in CKP mice linearly progressed with age and tumor burden while remaining absent in CKP2 mice (Fig. 1e, f, arrows), and were histologically confirmed metastases as indicated by the presence of pleomorphic nuclei (Fig. 1g). We also observed that CKP2 mice presented with less, and in most cases, no ascitic fluid volume, supporting our observation of diminished peritoneal metastases (Fig. 1h; Fig. S2C). Ctbp2 therefore drives primary PDAC tumor growth, and is, at least in part, a key dependency for metastasis in the CKP PDAC model.

**Fig. 2:**
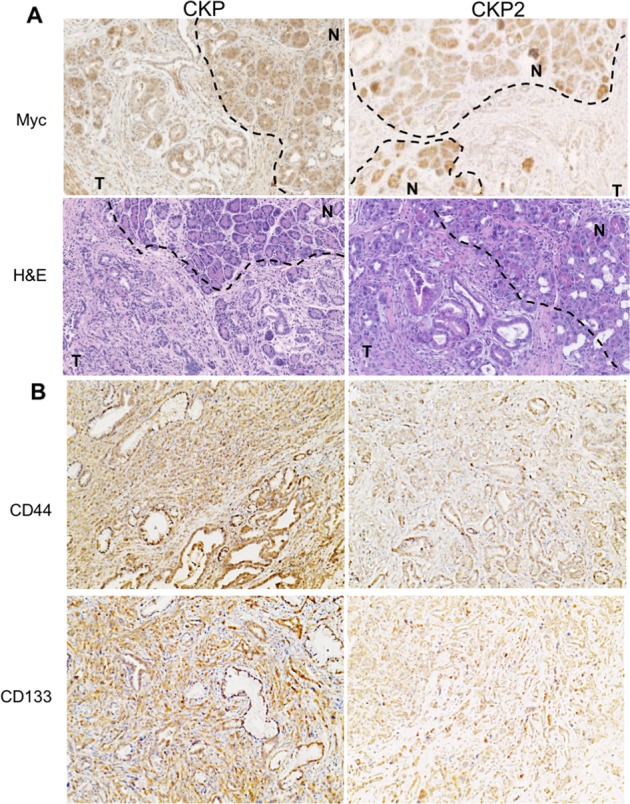
Expression of pancreatic TIC markers requires physiologic levels of Ctbp2. **a** Representative IHC analysis of c-Myc expression in CKP and CKP2 mouse pancreatic tumors; the dashed line divides the area of normal acini (N) from tumor (T). **b** Representative IHC analysis of cancer stem cell markers CD44 and CD133 in CKP and CKP2 mouse pancreatic tumors. Methods: For IHC of mouse pancreatic tumors from CKP and CKP2 mice refer to Fig. 1 IHC methods. The primary antibodies were used at a dilution of 1:100 in blocking buffer and incubated overnight. Antibodies—c-Myc (N-262, Cat no. sc-764, Santa Cruz Biotechnology), CD44 (Cat no. ab157107, Abcam, Cambridge, MA), and CD133 (Cat no. 18470-1-AP, Proteintech, Rosemont, IL)

The c-Myc proto-oncogene is required for the maturation and maintenance of embryonic and adult normal pancreatic acinar cells^17^. In K-RAS-driven PDAC, c-Myc also drives acinar-to-ductal metaplasia (ADM), a key step in pancreatic oncogenesis, and in the absence of a wild-type c-Myc gene, CKP mice develop only PanIN precancerous lesions, suggesting a prominent role for c-Myc in tumor progression^17^. More importantly, c-Myc also controls the generation of TIC-equivalent, self-renewing metastatic PDAC cells, and its overexpression in pancreatic progenitors led to PDAC, along with metastasis to the liver^17^. Given the key role of c-Myc in pancreatic oncogenesis and metastasis, and our findings that Ctbp2 modulates both tumor growth and metastasis in PDAC, we assessed c-Myc expression in CKP vs. CKP2 tumors by IHC. Comparing the expression of c-Myc in normal pancreatic acini (CKP and CKP2 pancreata have very few identifiable normal ducts; thus, acini are the only normal cells adjacent to tumor cells) vs. tumor cells in each genotype by IHC, CKP tumors stained moderately for c-Myc in both normal (2+; arrows) and malignant cells (2+; Fig. 2a, left), while Ctbp2-deficient CKP2 tumors maintained similar c-Myc staining to CKP pancreata in normal pancreatic acini (3+), but demonstrated weak, barely detectable, c-Myc staining (1+) in adjacent tumor cells (Fig. 2a, right). Overall, c-myc H-score analysis revealed significantly less-intense c-Myc staining in CKP2 tumors, as compared with CKP tumors (H scores of 25 vs. 125, respectively; *n* = 3; Fig. S2D). Thus, full Ctbp2 gene dosage is required for full expression of c-Myc in transformed pancreatic ductal cells in the CKP model. Our findings align with prior work in prostate cancer, where c-Myc was dependent on CtBP2 expression^18^, and reveal that c-Myc could therefore be an important downstream effector of the CtBP2 oncogenic transcriptional program, whether the mechanism is indirect or through direct transcriptional coactivation of c-Myc by CtBP2^18^.

Increasing evidence in colon and breast cancer models suggests that the CtBP2 transcriptional corepressor plays a key role in TIC formation and activity^3,11^. However, the linkage of CtBP2 to pancreatic TICs is entirely unexplored. CD133 is expressed on pancreatic cancer TICs, whereas CD44 is a TIC marker used in the assessment of most solid tumors^19,21^. Therefore, we sought to determine the expression of TIC markers CD133 and CD44 in CKP and CKP2 pancreatic tumors to understand stemness as it relates to *Ctbp2* allelic status in PDAC development. IHC analysis of tumors obtained from CKP2 mice revealed loss of expression of both CD44 as well as CD133, as compared with their expression in CKP tumors (Fig. 2b). Thus, Ctbp2 drives, or is at least required for, tumor stemness in PDAC, and the lack of stemness in CKP2 tumors may be linked to the observed lack of metastases (Fig. 1e–h), given the known role of TICs in metastatic seeding^19^.

**Fig. 3:**
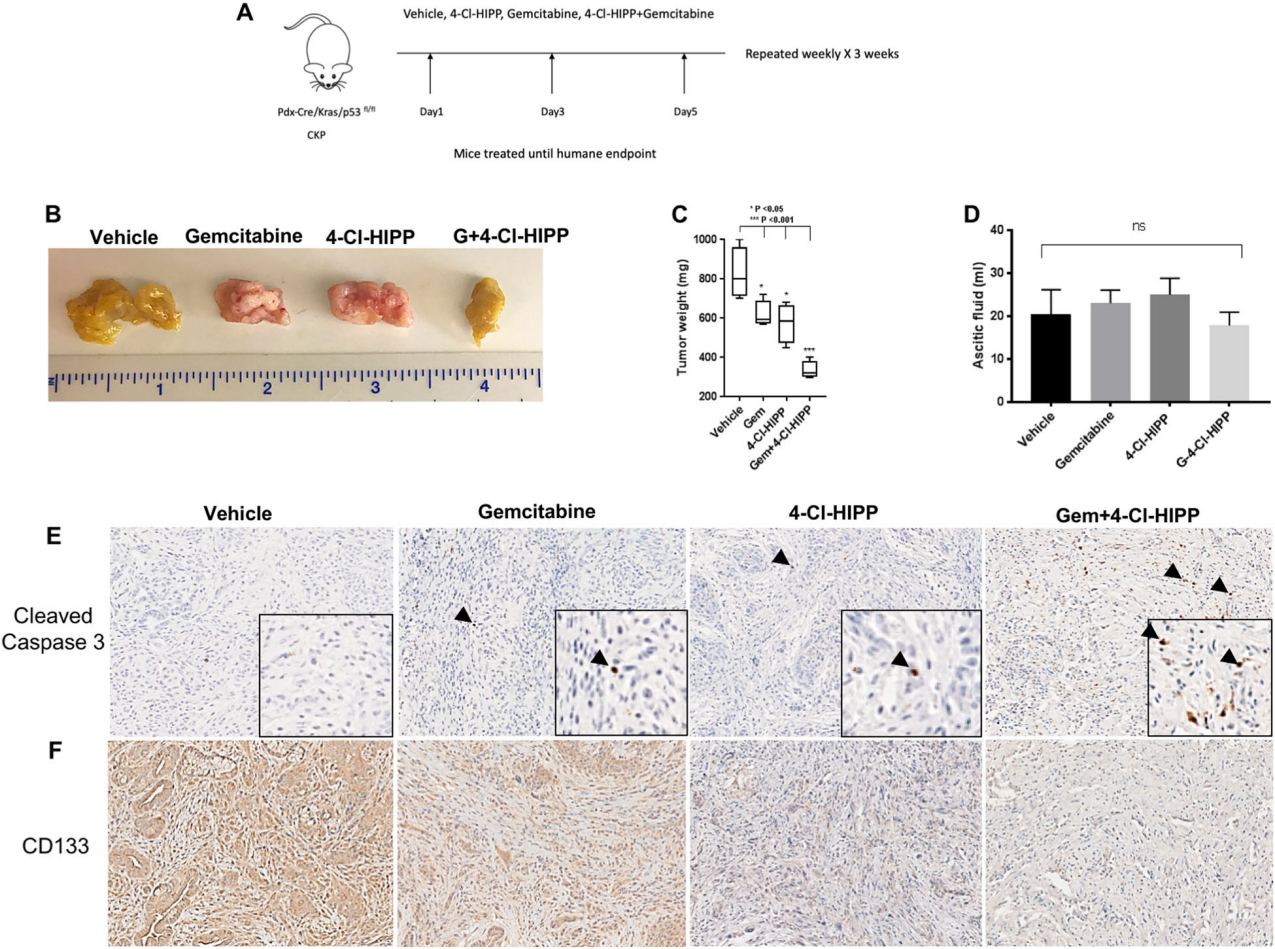
Combination therapy with CtBP2 inhibitor induces apoptosis and significantly reduces pancreatic tumor burden. **a** Schema for treatment of CKP mice with vehicle, gemcitabine, 4-Cl-HIPP, or the combination, 3× per week for 3 weeks or until humane endpoint. **b** Representative images of tumors obtained from CKP mice treated with vehicle, gemcitabine, 4-Cl-HIPP, or gemcitabine and 4-Cl-HIPP. **c** Quantification of tumor weights obtained from mice treated with above-listed drugs, *n* = 5 mice/treatment group. **d** Quantification of ascites aspirated from CKP mice treated with above-mentioned drugs, *n* = 5 mice/group. **e**, **f** Representative IHC staining of pancreatic tumors for TIC marker CD133 (**e**) and apoptosis indicator cleaved caspase 3 (**f**) obtained from CKP mice treated with above-mentioned drugs. Methods: CKP mice were injected with vehicle, gemcitabine (10 mg/kg, IP), 4-Cl-HIPP (100 mg/kg, IP) or a combination of both, intraperitoneally three times a week for 3 weeks, or until humane endpoint. At killing, tumor weights and ascites were measured. Refer to Fig. 1 Methods for IHC details; CD133 staining is detailed in Fig. 2; cleaved caspase 3 antibody (dilution 1:200; Cat no. 9661 Cell Signaling, Danvers, MA)

Given CtBP’s emerging role as a drug target^3^ and key role in PDAC tumor growth, metastasis, and stemness, we next sought to study the effect of the small-molecule CtBP inhibitor 4-Cl-HIPP^12^ in the CKP PDAC model, and determine if anti-CtBP therapy can also synergize with standard PDAC chemotherapy (gemcitabine)^20^. For this purpose, we treated CKP mice with vehicle, gemcitabine, 4-Cl-HIPP, or both agents for 3 weeks or till humane endpoint (Fig. 3a). Though both gemcitabine and 4-Cl-HIPP modestly decreased tumor burden by ~25% (*p* < 0.05), the combination of gemcitabine and 4-Cl-HIPP dramatically reduced primary tumor burden by >50% (Fig. 3b, c). Such a robust attenuation of tumor burden by the combination is highly significant, as PDAC in CKP mice begins essentially at birth, and drug cannot be administered till weaning at 6 weeks. Unlike what we observed with CKP2 vs. CKP mice, however, there was no change in ascitic fluid volume obtained from mice treated with drugs alone, or in combination (Fig. 3d). This may be due to peritoneal metastases already being well established by weaning in CKP mice (Fig. 1e), and well before drugs can be started that might interfere with ascites driven by peritoneal tumor implants. In addition, as might be expected by the lack of difference in metastasis between treatments, survival analysis of mice treated with vehicle, gemcitabine, 4-Cl-HIPP, or the combination revealed no statistically significant improvement in overall survival between drug treatment groups and vehicle treatment (Fig. S3A).

To understand the mechanism of 4-Cl-HIPP tumor inhibition and develop a pharmacodynamic marker for its activity, as CtBP is a well-known suppressor of pro-apoptotic factors^4^, we performed IHC analysis of tumors obtained from the above-mentioned treatment groups for the apoptosis marker cleaved caspase 3 (Fig. 3e; Fig. S3B). As expected based on the modest effect on tumor size, treatment with either gemcitabine or 4-Cl-HIPP alone, did not induce a statistically significant increase in apoptosis as determined by quantifying cleaved caspase 3-positive cells. Strikingly, however, tumors treated with gemcitabine/4-Cl-HIPP combination exhibited a synergistic and robust apoptotic response (5-fold over vehicle; *p* < 0.05; Fig. 3e; Fig. S3B).

We also explored expression of the TIC marker CD133 in drug-treated tumors, as a separate pharmacodynamic marker that could be correlated with not only decreased tumor burden, but also propensity to metastasis^21^. When comparing the expression of CD133 in tumors derived from vehicle, gemcitabine, 4-Cl-HIPP, or combination-treated mice, tumors derived from mice treated with 4-Cl-HIPP, or the combination, exhibited a robust and statistically significant ablation in CD133 expression (3-fold and 4-fold decrease, respectively; *p* < 0.05) as compared with the effect of gemcitabine, which alone had absolutely no effect on CD133 expression (Fig. 3f; Fig. S3C). These data showing a robust inhibition of CD133 by 4-Cl-HIPP are thus consistent with the role of Ctbp2 in promoting TIC activity in PDAC as seen in Fig. 2.

Modeling the combined effects of gemcitabine and 4-Cl-HIPP in human PDAC cells, we also treated the human pancreatic cancer cell line Panc-1 with vehicle, gemcitabine, 4-Cl-HIPP, or gemcitabine/4-Cl-HIPP, and observed a similar effect on induction of apoptosis as measured by immunoblot analysis of PARP cleavage and cleaved caspase 3, as seen in mouse tumors (Fig. S3D), which suggests that our findings are translatable from mouse PDAC into the human disease. Overall, these findings suggest that combined 4-Cl-HIPP/gemcitabine therapy was efficient at induction of tumor cell apoptosis in primary PDAC tumors, and effectively ablated tumor stemness potential as well, even if survival was not impacted due to the aggressive nature of the model.

The need for novel treatment paradigms for PDAC based on insights into mechanistic dependencies is of growing importance, given the lack of recurring easily targetable mutations. Our novel finding that CtBP1/2 are universally highly expressed in stage II–IV human PDAC, and that *Ctbp2* haploinsufficiency, modestly, but significantly, prolonged survival in the aggressive CKP mouse pancreatic cancer model, and more importantly rendered a metastasis-free phenotype, reveals CtBP to be a dependency and target in PDAC important for both primary tumor progression and the metastatic process. Whether the lack of CtBP1 expression in stage 1 tumors is of mechanistic or biologic significance will require further study of a larger sample size of stage 1 tumors, as due to the small number of stage 1 tumors in this study, no firm conclusion can be drawn that the generally improved prognosis in stage 1 PDAC can be attributed to the low expression of CtBP1.

Targeting the CtBP dehydrogenase with 4-Cl-HIPP modestly impacted primary tumor growth but robustly reduced CD133 TIC marker expression in a manner similar to genetic *Ctbp2* loss, with the exciting possibility that anti-CtBP therapy may preferentially target TIC populations within PDAC tumors. Combining 4-Cl-HIPP with the PDAC standard-of-care chemotherapeutic gemcitabine led to dramatic reductions in tumor size compared with either agent alone, and moreover, led to a synergistic induction of tumor cell apoptosis when neither agent alone caused any significant apoptotic induction. Anti-CtBP pharmacologic therapy was less effective than *Ctbp2* genetic knockout at limiting metastasis and prolonging survival in CKP mice, we presume, due to the inability to deliver drug therapy until the metastatic process was well under way by the time of weaning.

Further evaluation of 4-Cl-HIPP and related inhibitors, alone or in combination with standard therapies, is warranted in PDAC. Our data also suggest that anti-CtBP therapy, in general, may serve as both a novel anti-TIC therapy in settings where TICs represent a mechanism for cancer relapse and/or chemoresistance^22^, and also an indirect means of targeting c-Myc in the many Myc-dependent human cancers^17^.

### Study approval

Human PDAC specimens were obtained from the VCU Department of Pathology under an Institutional Review Board approved protocol (# HM20008572). All animal studies performed for this paper were approved by the VCU Institutional Animal Care and Use Committee (Approval # AD10001828).

### Statistics

Comparison between two groups was performed by using paired and unpaired student’s *t* tests and GraphPad Prism 7.0 software (GraphPad Software, San Diego, CA). A *P* value of less than 0.05 was considered significant. All error bars represent SEM. Kaplan–Meier analyses of mouse survival were performed by log-rank test in GraphPad Prism 7.0 software. The effect of CtBP1/2 on survival in the human tumor study was tested by using a Cox proportional hazards model for each gene separately by using the mean Allred score from the two reviewers as a predictor of survival, while also controlling for age at diagnosis, race, and sex. Human PDAC survival analyses were performed by using R 3.4.0.

## Supplementary information

Tables S1-S2; Figures S1-S3
