# Supplementary material for: CtBP—a targetable dependency for tumor-initiating cell activity and metastasis in pancreatic adenocarcinoma
Source: Oncogenesis. 2019 Oct 4;8(10):55. doi: 10.1038/s41389-019-0163-x (PMC6778071; doi:10.1038/s41389-019-0163-x)
Supplement: Supplementary file 1 — Tables S1-S2; Figures S1-S3 [file 41389_2019_163_MOESM1_ESM.pptx]

## Slide 1
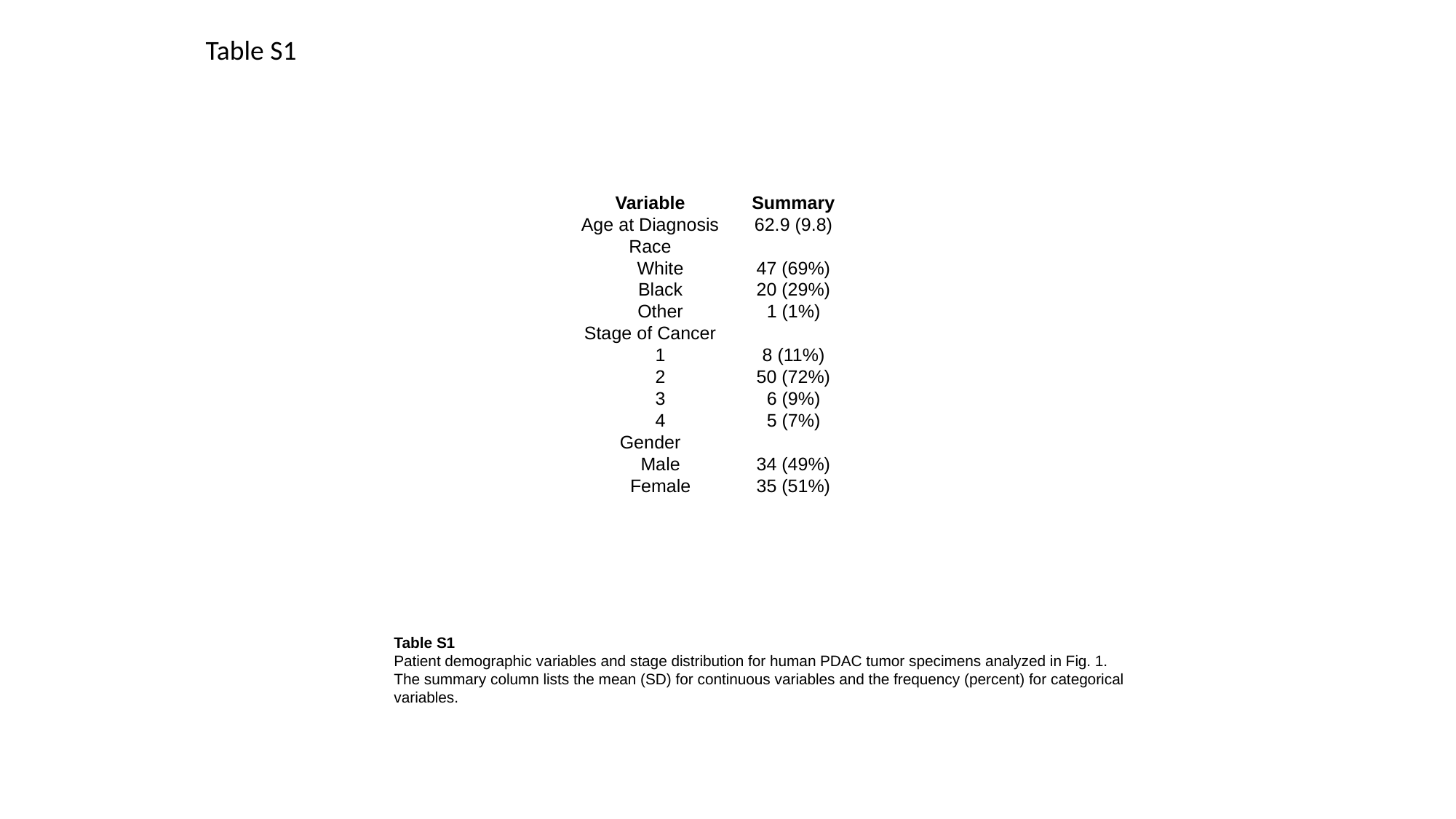

Table S1
| Variable | Summary |
| --- | --- |
| Age at Diagnosis | 62.9 (9.8) |
| Race | |
| White | 47 (69%) |
| Black | 20 (29%) |
| Other | 1 (1%) |
| Stage of Cancer | |
| 1 | 8 (11%) |
| 2 | 50 (72%) |
| 3 | 6 (9%) |
| 4 | 5 (7%) |
| Gender | |
| Male | 34 (49%) |
| Female | 35 (51%) |
Table S1
Patient demographic variables and stage distribution for human PDAC tumor specimens analyzed in Fig. 1. The summary column lists the mean (SD) for continuous variables and the frequency (percent) for categorical variables.

## Slide 2
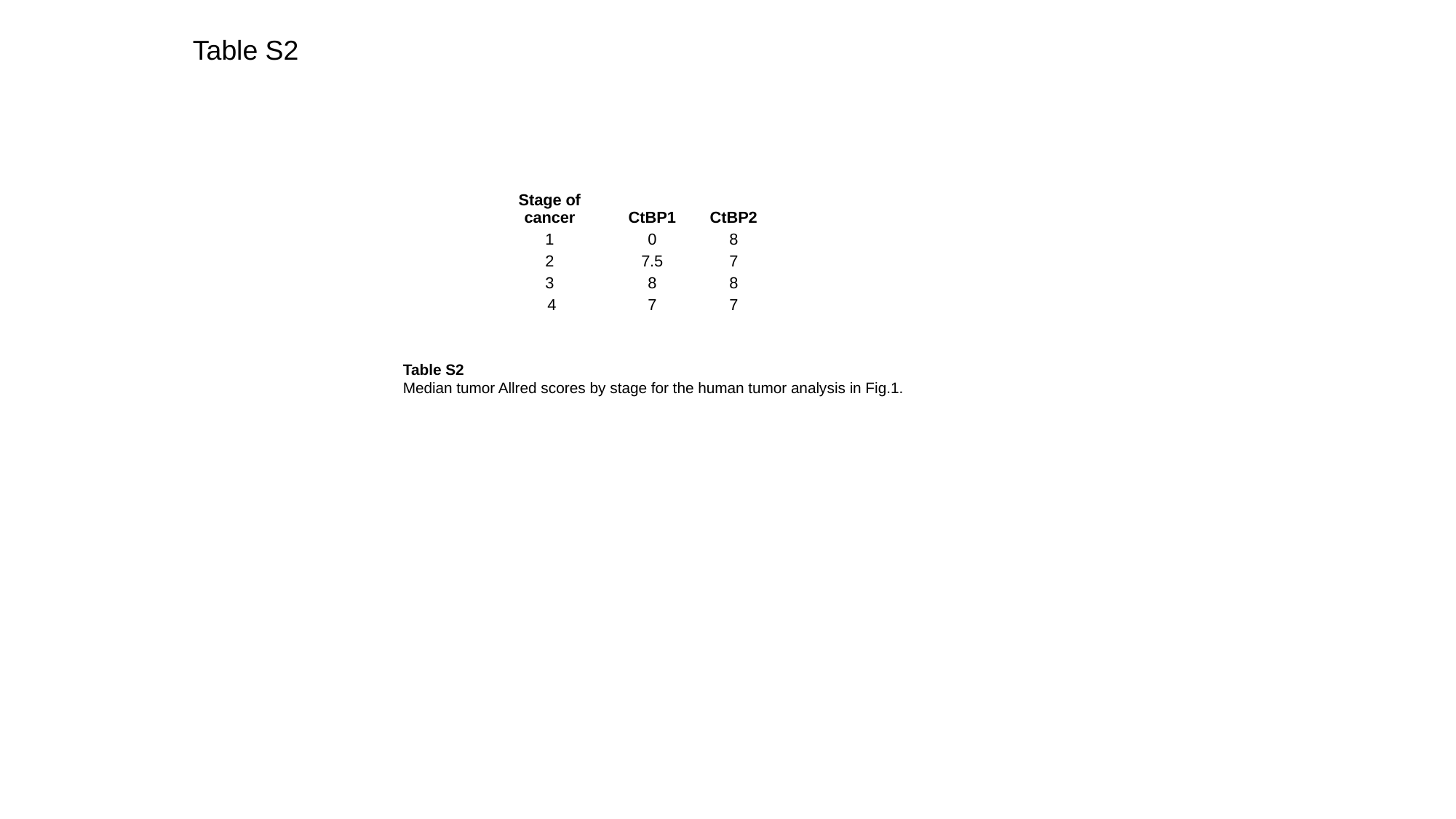

Table S2
| Stage of cancer | CtBP1 | CtBP2 |
| --- | --- | --- |
| 1 | 0 | 8 |
| 2 | 7.5 | 7 |
| 3 | 8 | 8 |
| 4 | 7 | 7 |
Table S2
Median tumor Allred scores by stage for the human tumor analysis in Fig.1.

## Slide 3
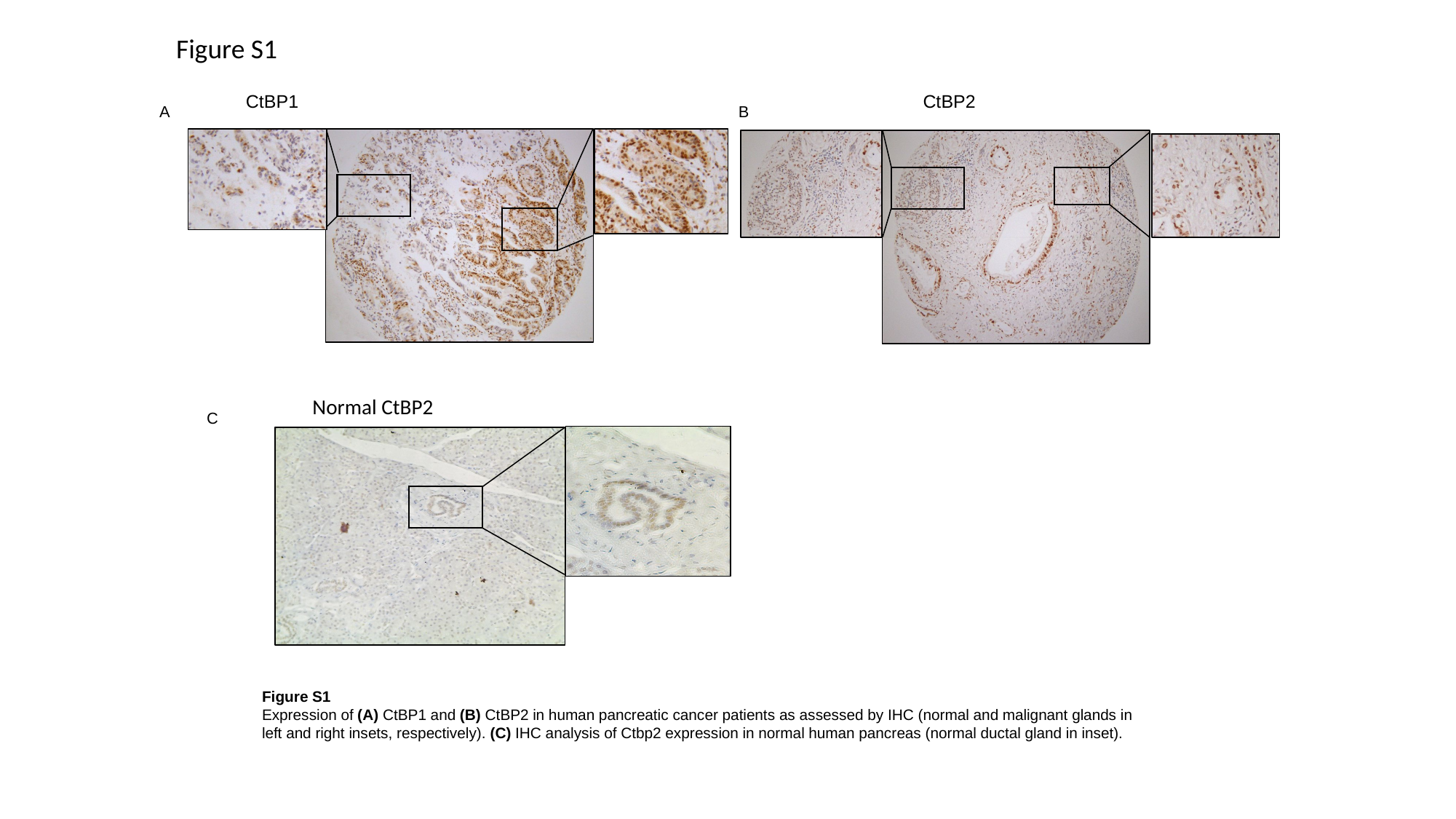

Figure S1
CtBP1
CtBP2
A
B
Normal CtBP2
C
Figure S1
Expression of (A) CtBP1 and (B) CtBP2 in human pancreatic cancer patients as assessed by IHC (normal and malignant glands in left and right insets, respectively). (C) IHC analysis of Ctbp2 expression in normal human pancreas (normal ductal gland in inset).

## Slide 4
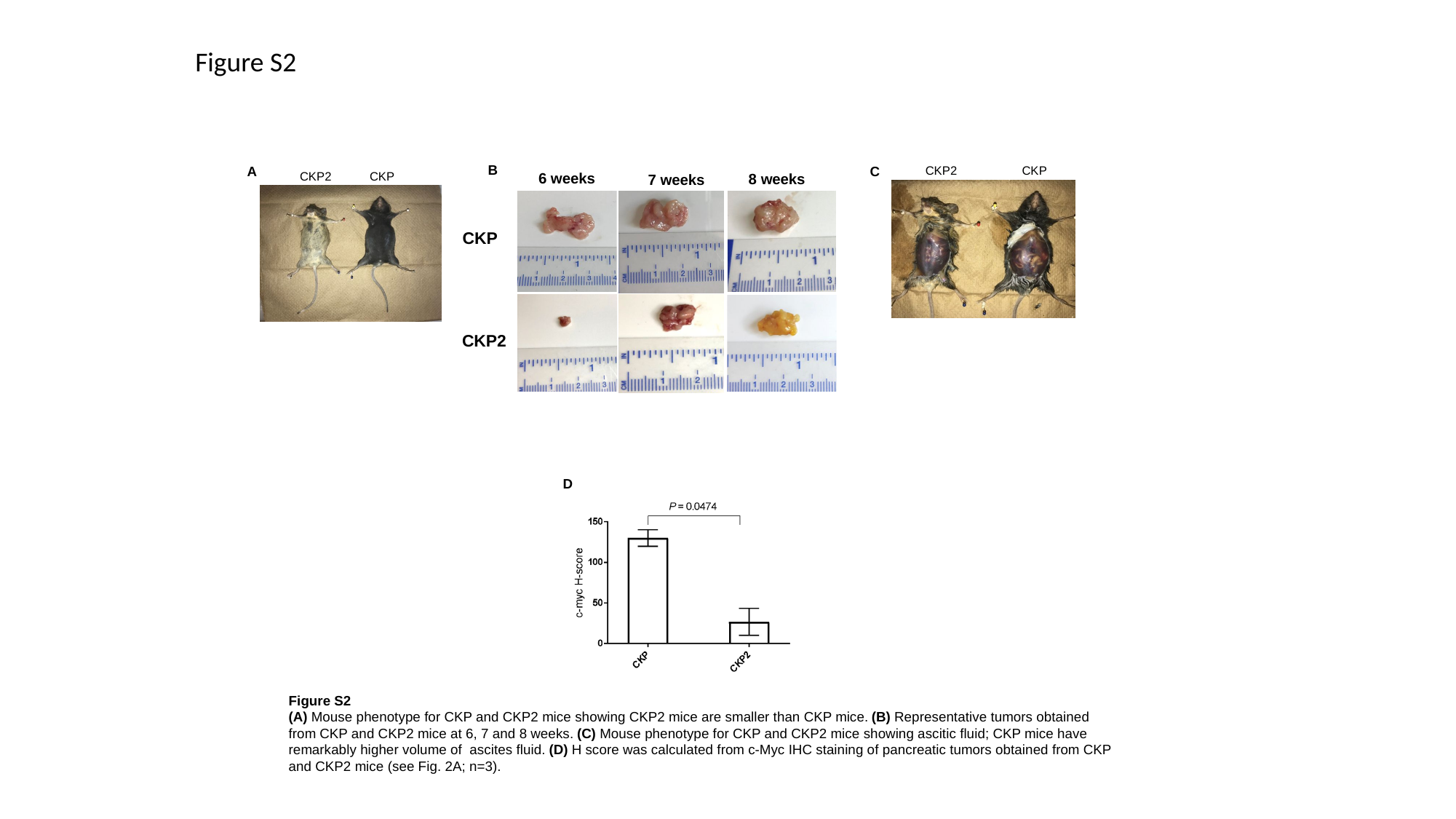

Figure S2
B
A
C
CKP2
CKP
CKP2
CKP
6 weeks
8 weeks
7 weeks
CKP
CKP2
D
Figure S2
(A) Mouse phenotype for CKP and CKP2 mice showing CKP2 mice are smaller than CKP mice. (B) Representative tumors obtained from CKP and CKP2 mice at 6, 7 and 8 weeks. (C) Mouse phenotype for CKP and CKP2 mice showing ascitic fluid; CKP mice have remarkably higher volume of ascites fluid. (D) H score was calculated from c-Myc IHC staining of pancreatic tumors obtained from CKP and CKP2 mice (see Fig. 2A; n=3).

## Slide 5
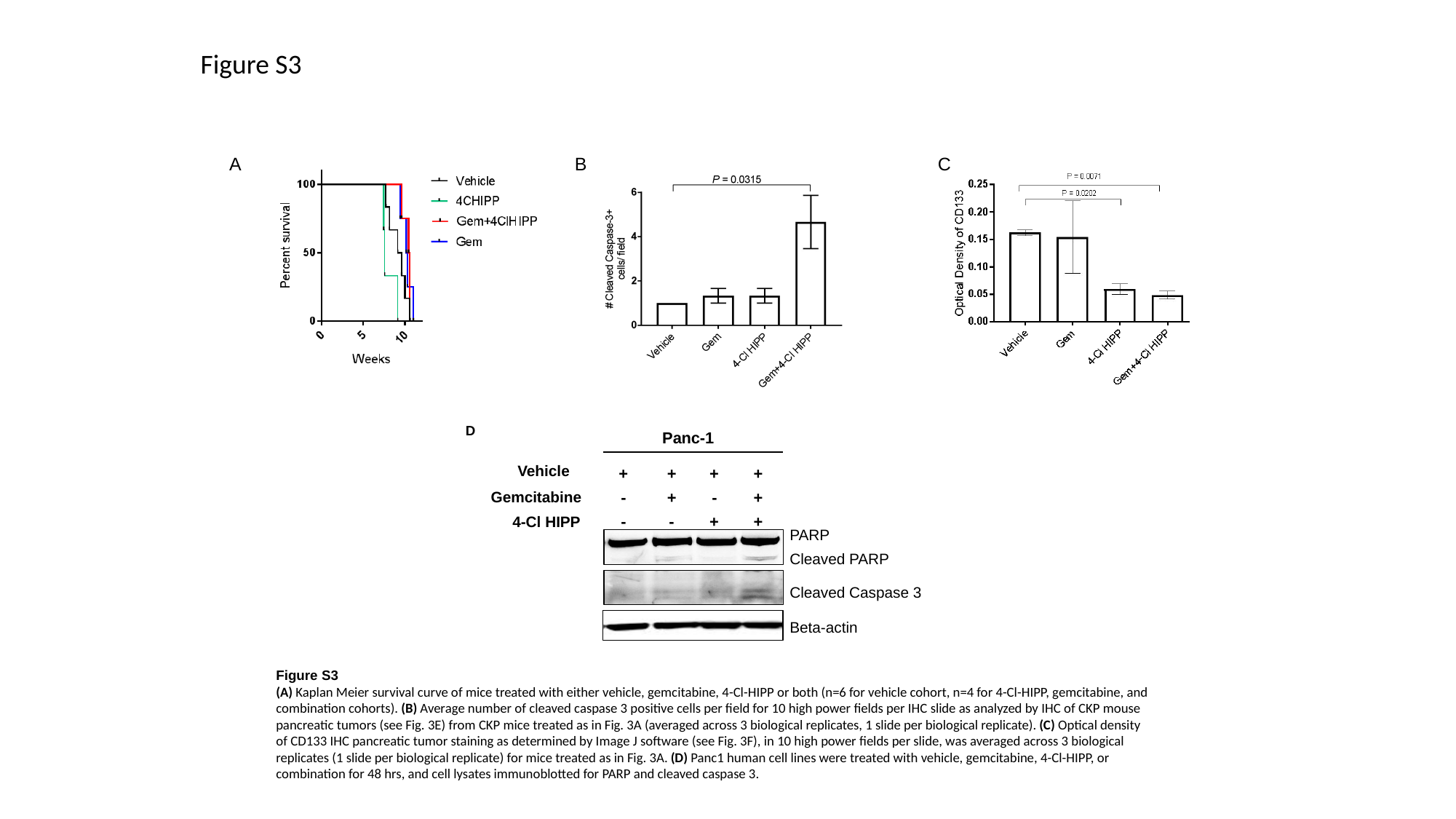

Figure S3
A
B
C
D
Panc-1
 Vehicle
 +
+
 +
 +
 Gemcitabine
 -
+
 -
 +
 -
 +
 +
 -
 4-Cl HIPP
PARP
Cleaved PARP
Cleaved Caspase 3
Beta-actin
Figure S3
(A) Kaplan Meier survival curve of mice treated with either vehicle, gemcitabine, 4-Cl-HIPP or both (n=6 for vehicle cohort, n=4 for 4-Cl-HIPP, gemcitabine, and combination cohorts). (B) Average number of cleaved caspase 3 positive cells per field for 10 high power fields per IHC slide as analyzed by IHC of CKP mouse pancreatic tumors (see Fig. 3E) from CKP mice treated as in Fig. 3A (averaged across 3 biological replicates, 1 slide per biological replicate). (C) Optical density of CD133 IHC pancreatic tumor staining as determined by Image J software (see Fig. 3F), in 10 high power fields per slide, was averaged across 3 biological replicates (1 slide per biological replicate) for mice treated as in Fig. 3A. (D) Panc1 human cell lines were treated with vehicle, gemcitabine, 4-Cl-HIPP, or combination for 48 hrs, and cell lysates immunoblotted for PARP and cleaved caspase 3.
